# Publisher Correction: Nanoplasmonic electron acceleration by attosecond-controlled forward rescattering in silver clusters

**DOI:** 10.1038/s41467-018-02903-y

**Published:** 2018-02-07

**Authors:** Johannes Passig, Sergey Zherebtsov, Robert Irsig, Mathias Arbeiter, Christian Peltz, Sebastian Göde, Slawomir Skruszewicz, Karl-Heinz Meiwes-Broer, Josef Tiggesbäumker, Matthias F. Kling, Thomas Fennel

**Affiliations:** 1 Institut für Physik, Universität Rostock, Albert-Einstein-Str. 23, D-18059 Rostock, Germany; 2 Physik Department, Ludwig-Maximilians-Universität München, Am Coulombwall 1, D-85749 Garching, Germany; 3 Max-Planck Institut für Quantenoptik, Hans-Kopfermann-Straße 1, D-85748 Garching, Germany; 4 Max-Born-Institut für Nichtlineare Optik und Kurzzeitspektroskopie, Max-Born-Straße 2A, D-12489 Berlin, Germany

Correction to: *Nature Communications* 10.1038/s41467-017-01286-w, published online 30 October 2017

The original PDF version of this Article contained an error in Equation 1. The original HTML version of this Article contained errors in Equation 2 and Equation 4. These errors have now been corrected in both the PDF and the HTML versions of the Article.

The original PDF version of this Article contained an error in Equation 1. A dot over the first occurrence of the variable **r**_*i*_ was missing, and incorrectly read:

$$E_{{\mathrm{sp}}} = \frac{m}{2}\left| {{\mathbf{r}}_i} \right|^2 + V\left( {{\mathbf{r}}_i\left( t \right),t} \right)$$

The correct form of Equation 1 is as follows:

$$E_{{\mathrm{sp}}} = {\frac{m}{2}}\left| {\dot{\mathbf {r}}}_{i} \right|^{2} + V\left( {\mathbf{r}}_{i}\left( t \right),t \right)$$

This has now been corrected in the PDF version of the Article. The HTML version was correct from the time of publication.

The original HTML version of this Article contained errors in Equation 2 and Equation 4.

In Equation 2, a circle over the first occurrence of the variable **r**_*i*_ replaced the intended dot, and incorrectly read:

$$\dot E_{{\mathrm{sp}}} = q_i {{\mathop {\bf r}\limits^{\circ}}_i} (t)\cdot{\cal E}_{{\mathrm{las}}} + \frac{\partial }{{\partial t}}V\left( {{\mathbf{r}}_{i}\left( t \right),t} \right)$$

The correct form of Equation 2 is as follows:

$$\dot E_{\mathrm{sp}} = q_i{\dot{\mathbf {r}}}_{i}(t)\cdot{\cal E}_{\mathrm{las}} + \frac{\partial }{\partial t}V\left( {\mathbf{r}}_{i}(t),t \right)$$

In Equation 4, circles over the first and fifth occurrences of the variable **r**_*i*_ replaced the intended dots, and incorrectly read:

$$\frac{\mathrm{d}}{{{\mathrm{d}}t}}E_{\mathrm{sp}} = m_{i}{{\mathop {\bf r}\limits^ \circ}_{i}} \cdot {\ddot{\mathbf{r}}}_{i} + \left[ {\nabla _{{\mathbf{r}}_{i}}V\left( {{\mathbf{r}}_{i},t} \right)} \right].{{\mathop {\bf r}\limits^ \circ}_{i}} + \frac{\partial }{{\partial t}}V\left( {{\mathbf{r}}_{i},t} \right)$$

The correct form of Equation 4 is as follows:

$$\frac{{\mathrm{d}}}{{{\mathrm{d}}t}}E_{{\mathrm{sp}}} = m_{i}{\dot{\mathbf {r}}}_{i}.{\ddot{\mathbf {r}}}_{i} + \left[ {\nabla _{{\mathbf{r}}_{i}}V\left( {{\mathbf{r}}_{i},t} \right)} \right].{\dot{\mathbf {r}}}_{i} + \frac{\partial }{{\partial t}}V\left( {{\mathbf{r}}_{i},t} \right)$$

This has now been corrected in the HTML version of the Article. The PDF version was correct from the time of publication.

